# Towards anti-influenza nasal sprays: infection models and opportunities to reduce animal testing

**DOI:** 10.1038/s44298-026-00255-9

**Published:** 2026-11-02

**Authors:** Jolly Thomas, Christoph André Nilius, Leon Matis, Sabrina Schnur, Anja Himmelstein, Armin Walter Novak, Jens Kurreck, Marius Hittinger, Daniel Christian Lauster

**Affiliations:** 1 Freie Universität Berlin, Institute of Pharmacy, Biopharmaceuticals, Berlin, Germany; 2 University of Applied Sciences Kaiserslautern, Zweibrücken, Germany; 3 Saarland University, Department of Pharmacy, Biopharmaceutics and Pharmaceutical Technology, Saarbrücken, Germany; 4 MucosaTec GmbH, Schiffweiler, Germany; 5 PharmaScienceHub, Saarbrücken, Germany; 6 Technische Universität Berlin, Institute for Biotechnology, Applied Biochemistry, Berlin, Germany; 7 PharmBioTec GmbH, Schiffweiler, Germany

**Keywords:** Biotechnology, Diseases, Drug discovery, Medical research, Microbiology

## Abstract

The nasal mucosa is an important site of influenza virus entry and early replication, yet approved antivirals are not designed for nasal delivery. This Review summarizes antiviral strategies suitable for nasal administration and evaluates animal and human-relevant models for their development. We discuss route-specific formulation and regulatory requirements and propose a translational framework integrating in vitro, ex vivo and microphysiological systems with targeted in vivo studies to reduce animal use.

## Introduction

Influenza is a respiratory infection caused by influenza viruses and occurs seasonally in temperate regions during the colder months of the year^1^. Every year, seasonal influenza affects approximately one billion people worldwide and results in 3–5 million severe respiratory infections, causing an estimated 290,000–650,000 deaths^2^. The disease burden is unevenly distributed; low and middle-income countries (LMICs) account for approximately 83% of annual influenza-related respiratory deaths worldwide, with the highest mortality observed in sub-Saharan Africa and South/Southeast Asia^3^.

At the regional level, epidemiological patterns vary considerably. In Germany, influenza surveillance conducted by the Robert Koch Institute (RKI) shows that seasonal influenza activity varies between epidemic waves and is associated with a substantial burden of severe disease, with hospitalizations and severe outcomes occurring predominantly in older age groups^4^. In Asia, influenza epidemiology shows considerable regional variability, with differences in transmission patterns and seasonality between temperate and tropical regions^5^. Age is a critical determinant of disease severity, with elderly individuals and young children facing the highest risks of hospitalization and mortality^6^. Among children under 5 years, the burden is particularly pronounced in low-income and lower-middle-income countries, which accounted for approximately 82% of in-hospital deaths from influenza-associated acute lower respiratory infections (ALRIs) in 2018^7^. Collectively, these factors underscore influenza as a major global public health challenge that demands effective preventive and therapeutic strategies.

Influenza viruses are primarily transmitted via respiratory aerosols generated during sneezing, coughing, talking, or breathing^8^. The airborne route represents the predominant mode of transmission, as virus-containing liquid aerosols can remain infectious for extended periods and facilitate long-range human-to-human spread^9^. Larger droplets (size > 5 µm) typically settle within one to two meters and contribute mainly to droplet or contact-based transmission, whereas smaller aerosols (<5 µm) can remain airborne for prolonged periods depending on environmental conditions^10^. An Influenza infection can range from asymptomatic or mild disease progression to severe respiratory illness. Human challenge studies have demonstrated that even low infectious doses of an aerosolized influenza virus can induce clinical symptoms such as fever and cough^11,12^. Similar findings have been observed in ferret models, supporting the high infectivity in the upper respiratory tract and airborne transmission of influenza viruses^13^.

Virus-containing aerosols deposit on the nasal and nasopharyngeal mucosa, making the nasal cavity a major site of initial contact between influenza viruses and the human host. Early viral replication within the nasal epithelium precedes and may promote subsequent dissemination to the trachea and bronchi and, in more severe cases, the alveolar compartment^14,15^. This upper-to-lower respiratory progression defines a critical therapeutic window during which local intervention at the nasal mucosa may prevent or limit disease progression before systemic or lower airway involvement is established. The nasal cavity therefore represents an important site for early antiviral intervention and provides the biological rationale for locally acting antiviral nasal spray strategies.

Influenza viruses belong to the *Orthomyxoviridae* family of enveloped, segmented, negative-strand RNA viruses and are classified into four types (A-D)^16^. Among these, influenza A and B viruses are responsible for the majority of human epidemics and pandemics^17^. The viral envelope contains the glycoproteins hemagglutinin (HA) and neuraminidase (NA), which play essential roles in virus entry and release from the host cell membrane^18,19^. HA mediates the attachment of viral particles to sialic acid (N-acetyl neuraminic acid) residues on host cells and enables membrane fusion during viral entry, whereas NA cleaves sialic acid (SA) residues to release newly formed virions^20^. Continuous genetic variation of HA and NA through antigenic drift and occasional antigenic shift allows influenza viruses to evade host immunity and contributes to the recurring nature of seasonal outbreaks^21^.

**Fig. 1:**
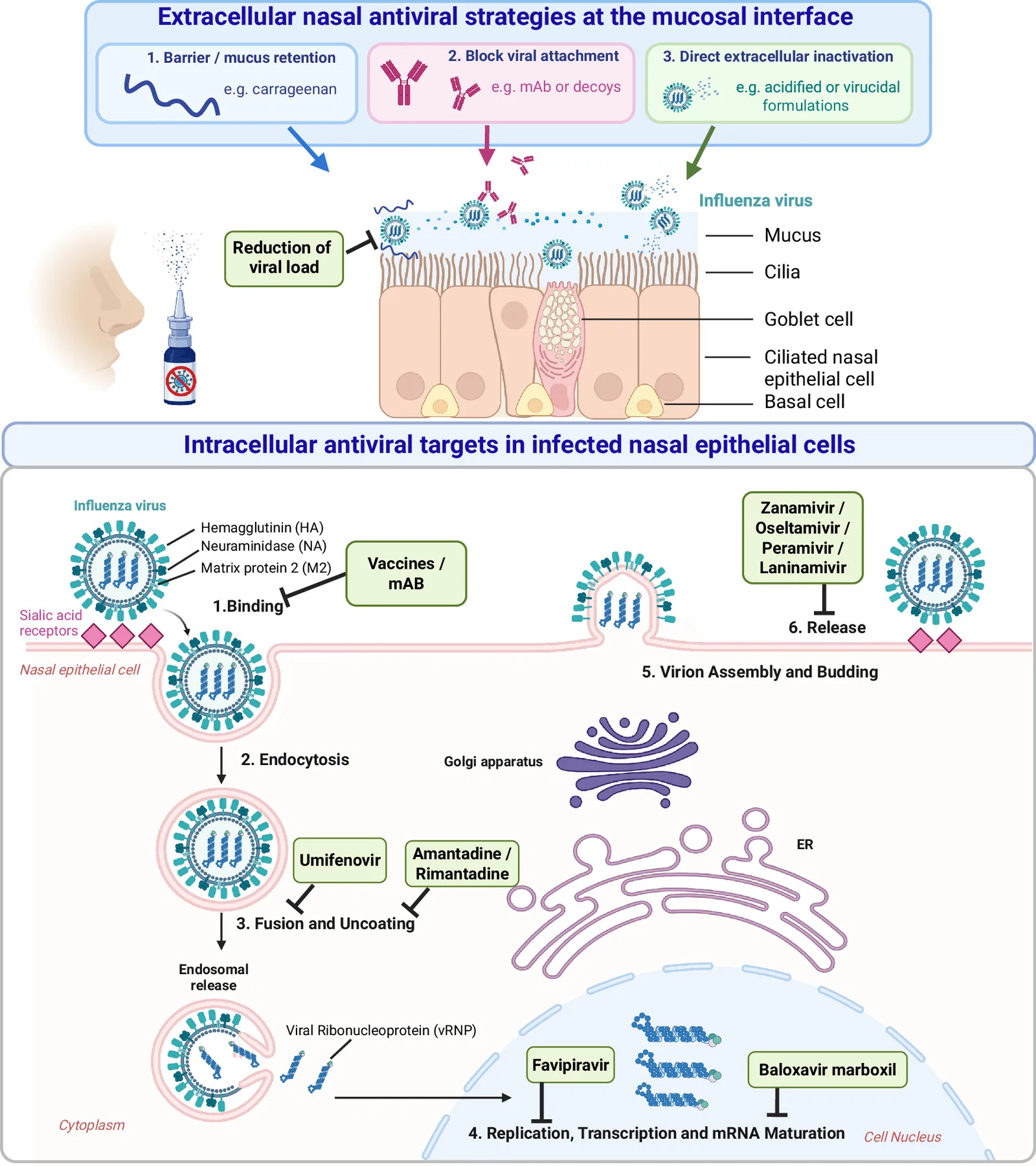
Influenza virus replication cycle and antiviral intervention strategies at the nasal epithelial interface. The upper panel illustrates extracellular antiviral strategies acting within the nasal mucus layer before viral entry into epithelial cells. These include barrier and mucus-retention approaches, exemplified by carrageenan; inhibition of viral attachment by neutralizing antibodies or receptor-mimicking decoys; and direct extracellular virus inactivation by acidified or virucidal formulations. These locally acting strategies aim to reduce the number and mobility of infectious virions reaching the nasal epithelial surface. The lower panel summarizes the influenza virus replication cycle and established antiviral intervention points in or at infected nasal epithelial cells. Influenza virus hemagglutinin (HA) binds to sialic acid receptors at the epithelial surface, followed by endocytosis, endosomal acidification, membrane fusion, and viral uncoating. Attachment can be inhibited by neutralizing antibodies, whereas umifenovir inhibits HA-mediated membrane fusion and the M2 ion-channel blockers amantadine and rimantadine interfere with viral uncoating. Viral ribonucleoprotein complexes are subsequently transported to the nucleus, where favipiravir and the cap-dependent endonuclease inhibitor baloxavir marboxil interfere with viral RNA replication and transcription. Following assembly and budding at the plasma membrane, progeny virions are released through neuraminidase-mediated cleavage of sialic acid residues, a process inhibited by neuraminidase inhibitors including oseltamivir, zanamivir, peramivir, and laninamivir. This image was created with BioRender.com.

A variety of antiviral strategies targeting different stages of the influenza virus life cycle have been developed, including inhibitors of viral entry, uncoating, genome replication, and virion release (Fig. 1). One important strategy focuses on blocking viral attachment and entry into host cells^22^. This can be achieved by using broadly neutralizing monoclonal antibodies targeting conserved regions of influenza HA, including the HA stem or receptor-binding region^23^. Such antibodies have demonstrated strong neutralizing activity and are currently the subject of intensive research, although no antibody-based therapy against the influenza A virus has yet reached market approval^24^. An alternative approach is based on mimicking host cell receptors. In this strategy, sialic acid moieties are presented on polymeric scaffolds or other multivalent structures, thereby acting as decoy receptors that block viral binding to host cells^25,26^. These receptor-mimicking systems are particularly attractive for topical applications at mucosal surfaces, where they can intercept viral particles before cellular infection occurs.

Beyond entry inhibition, several antiviral drugs target intracellular stages of the influenza virus replication cycle. Fusion of the viral envelope with the endosomal membrane can be blocked by umifenovir (arbidol), which stabilizes the prefusion conformation of HA and thereby inhibits the low pH-induced conformational changes required for membrane fusion^27^. The adamantanes amantadine and rimantadine inhibit the viral M2 proton channel, thereby interfering with endosomal acidification of the virion and subsequent viral uncoating^28,29^. Viral genome replication can be inhibited by compounds targeting the viral polymerase complex, including cap-dependent endonuclease inhibitors such as baloxavir marboxil and the RNA-dependent RNA polymerase inhibitor favipiravir. Finally, neuraminidase inhibitors such as oseltamivir, zanamivir, peramivir, and laninamivir prevent the release of newly formed virions from infected cells^30,31^. Resistance to these antivirals, however, often limits their efficacy. Additional therapeutic strategies under development include RNA interference (RNAi), antisense oligonucleotides (ASOs), and CRISPR/Cas13-based RNA targeting^32^, as well as host-directed approaches targeting cellular signaling pathways involved in influenza virus replication, including the PI3K/Akt and Raf/MEK/ERK pathways^33^. Innate immune modulators, including intranasal TLR2/6 and STING agonists, are being explored as host-directed antiviral approaches^34,35^.

Despite this broad range of antiviral concepts, currently approved influenza therapeutics are predominantly small-molecule drugs administered orally or intravenously, with zanamivir delivered by oral inhalation. None is specifically designed for local delivery to the nasal mucosa, an important site of viral entry and early replication^36^. Among the investigational concepts described above, entry inhibitors are particularly well suited to local nasal application because they can intercept viral particles at the mucosal surface before cellular infection occurs^37^. Because early nasal replication precedes subsequent spread to the lower respiratory tract, intervention during this window may help limit disease progression.

Current influenza prevention and treatment rely primarily on seasonal vaccination and, where appropriate, prophylactic or early therapeutic administration of antiviral drugs^16^. These approaches have proven effective in reducing disease severity and transmission but are generally not optimized to directly target the nasal epithelium as the initial site of infection. Local delivery of antiviral agents via nasal sprays could therefore provide several advantages, including direct targeting of the nasal mucosa, higher local drug concentrations, faster onset of action, and reduced systemic exposure^38^. In addition, nasal administration represents a non-invasive and patient-friendly route that may facilitate rapid and large-scale deployment during seasonal outbreaks or pandemics.

## Nasal anatomy and physiology relevant to intranasal drug delivery

The potential advantages of local antiviral treatment are closely linked to the anatomical and physiological characteristics of the nasal cavity. The nasal epithelium is not uniform, it consists of the anterior vestibule lined by squamous epithelium bearing vibrissae that filter larger inhaled particles before the airstream reaches the respiratory region. The respiratory region is the largest part of the cavity lined by pseudostratified ciliated columnar epithelium and mucus-secreting goblet cells. A smaller olfactory region at the roof of the cavity, together with nasal-associated lymphoid tissue near the nasopharynx, completes the anatomical subdivisions relevant to drug deposition^39,40^.

This respiratory epithelium is covered by a thin mucus layer, only a few micrometers thick that traps inhaled particles and pathogens and forms the first physical barrier to infection. Motile cilia propel this mucus layer posteriorly toward the nasopharynx at a rate of several millimeters per minute, a process termed mucociliary clearance that under normal conditions renews the nasal mucus layer roughly every 10–20 min^39–41^.

The same defense mechanisms that protect the respiratory tract therefore also create important barriers to local drug delivery. Rapid mucociliary clearance limits nasal residence time and consequently local bioavailability. In addition, the physicochemical properties of the nasal cavity, including the mildly acidic pH of nasal secretions (typically approximately 5.5–6.5 in healthy adults), mucus viscosity, and its comparatively small deliverable volume, constrain formulation design and must be considered when developing antiviral nasal sprays^40,41^.

These formulation constraints also determine what is required from an experimental model. Demonstrating antiviral activity alone is insufficient if the model does not reproduce the mucosal environment or the route by which the formulation will ultimately be administered. Animal models have traditionally played an important role in influenza research, but species-specific differences in respiratory physiology can limit their translational relevance and their use also raises ethical concerns.

In parallel, advances in human-relevant in vitro, ex vivo, and microphysiological systems provide new opportunities to investigate influenza infection and antiviral efficacy under more controlled conditions and physiologically relevant conditions^42,43^. Microphysiological systems are engineered in vitro platforms that reproduce selected aspects of organ- or tissue-level physiology by integrating living cells within controlled microfluidic environments. For nasal drug development, these approaches are particularly relevant because local interactions among the formulation, mucus layers, epithelial surface, and virus can directly determine therapeutic performance.

In this context, the present review aims to (i) discuss the biological rationale and current approaches for antiviral nasal spray strategies against influenza viruses and (ii) critically evaluate available experimental platforms, including animal models and emerging animal-free systems that can support the development and assessment of such formulations while contributing to the reduction of animal testing.

## Nasal antiviral formulations for prevention and early treatment of influenza

The nasal mucosa represents an attractive site for prophylactic and early antiviral intervention because it constitutes a major site of influenza virus entry and initial replication^14,15^. However, intranasal vaccination, locally acting antiviral formulations, and systemically administered influenza therapeutics should be distinguished because they differ substantially in their mechanisms of action, formulation requirements, and regulatory pathways^22,34,38^. The requirements for prophylactic and post-exposure nasal interventions also differ substantially. Prophylactic strategies can establish protection at the mucosal surface before viral exposure, whereas therapeutic nasal formulations must act during the comparatively short period in which viral replication remains concentrated in the upper respiratory tract^22^. Once infection has disseminated toward the lower respiratory tract, treatment restricted to the nasal cavity may no longer provide sufficient exposure at all infected sites^15,38^. This limited therapeutic window may partly explain why intranasal approaches have been more successful for prevention than for treatment and highlights the importance of early administration and sufficient local drug exposure for therapeutic nasal antivirals^22,38^. The clinical feasibility of intranasal vaccination against influenza is demonstrated by the live-attenuated influenza vaccines (LAIV), marketed as FluMist in the United States and Fluenz in Europe^44,45^. Although LAIV is a vaccine rather than an antiviral treatment its clinical use demonstrated that nasal administration is feasible and acceptable, particularly in children^46,47^.

Beyond vaccination, several locally acting antiviral concepts have been explored that intervene directly at the mucosal surface. Among non-vaccine approaches, iota-carrageenan, a highly sulfated galactan polysaccharide derived from red algae, has been evaluated in nasal spray formulations in patients with respiratory viral infections, including influenza-positive participants, and was associated with reductions in viral load and/or symptom duration^48^. Carrageenan acts predominantly through multivalent interactions with viral particles that inhibit their attachment to epithelial cells and therefore represents a locally acting, primarily virustatic barrier strategy rather than a conventional influenza antiviral drug. This distinction is also relevant from a regulatory perspective, because products acting predominantly through physical mechanisms may follow different development pathways from pharmacologically active medicinal products.

A more target-specific approach is provided by antiviral antibodies. Broadly neutralizing monoclonal antibodies targeting conserved regions of HA can inhibit receptor engagement and membrane fusion, whereas NA-directed antibodies primarily inhibit NA enzymatic activity, thereby restricting virion release and spread and, in some settings, interfering with viral entry^49^. Antibodies against both glycoproteins may additionally recruit Fc-mediated effector functions. Importantly, the concept of local antibody delivery has now progressed beyond experimental proof of principle. Intranasal administration of the broadly neutralizing HA-stem antibody CR9114 provided complete prophylactic protection against lethal H5N1 challenge in mice. More recently, first-in-human Phase 1 studies showed that intranasally administered CR9114 was safe and well tolerated and achieved functional antibody concentrations in nasal mucosal lining fluid, providing direct translational evidence for local antibody-based influenza prophylaxis^50,51^.

Antibody functionality can be further extended through molecular conjugation, although the relevance of individual approaches to nasal delivery differs considerably. Site-specific conjugation of zanamivir to the broadly neutralizing HA-stem antibody MEDI8852 generated a bifunctional antibody-drug conjugate combining HA targeting with NA inhibition against influenza A and B viruses. However, this conjugate was evaluated following systemic administration and therefore serves primarily as a proof of concept for multifunctional antiviral conjugates^52^. Similarly, CD388 is a long-acting zanamivir-based drug-Fc conjugate rather than an influenza-specific antibody and has been developed for systemic prophylaxis^53^. More directly relevant to the nasal route, an intranasally administered multivalent nanobody-drug conjugate carrying four zanamivir molecules (VHHκ–Zan₄) provided complete protection against lethal influenza challenge in mice, demonstrating the potential of locally delivered biologic small molecule conjugates^54^.

For locally administered antibodies, however, antiviral potency is only one determinant of efficacy; residence time within the rapidly cleared mucus layer is equally important. Influenza-binding antibodies have been shown to immobilize influenza virions in fresh human airway mucus, supporting an antibody-mediated muco-trapping mechanism that may promote clearance together with mucociliary transport^55^. Strategies that actively increase antibody-mucus interactions could therefore prolong the residence time of locally administered antibodies. Recently, antibody-Mucus Anchoring Protein conjugates based on the mucus-binding protein trefoil factor 3 (TFF3) enhanced antibody retention and reduced viral mobility in a SARS-CoV-2 model. Although this approach has not yet been evaluated against influenza, TFF3-mediated mucus anchoring could represent a transferable strategy for prolonging the residence time of anti-influenza antibodies at the nasal mucosa^56^.

Whereas antibody-based strategies act primarily by neutralizing extracellular virions or promoting their clearance at the mucosal surface, nucleic-acid-based therapeutics provide a complementary means of interfering with infection after cellular entry. Small interfering RNAs (siRNAs) can directly suppress viral replication by targeting conserved influenza virus transcripts. In vivo studies demonstrated that siRNAs targeting conserved regions of the influenza A virus nucleoprotein (NP) and polymerase acidic protein (PA) genes reduced lung viral titers and protected mice from lethal influenza challenge. In addition, Ge et al. showed that influenza-specific RNA interference reduced viral replication when administered before or after infection and that intranasal administration of DNA constructs expressing influenza-specific shRNAs also reduced viral titers in the lungs^57,58^. Their translation to nasal therapy is nevertheless limited by extracellular instability, intracellular delivery requirements, and potential innate immune activation, motivating the development of chemically modified RNAs and nanoparticle carriers^59^. In contrast, mRNA approaches generally act indirectly by expressing therapeutic proteins or modulating the host response rather than by targeting the viral genome itself. The IL-35 mRNA/LNP example described previously was developed for influenza-associated lung inflammation rather than specifically for nasal administration and should therefore be regarded as a host-directed proof of concept rather than direct evidence for an antiviral nasal spray^60^.

Beyond sequence-specific nucleic-acid therapeutics, polymeric and supramolecular materials offer an extracellular route to antiviral activity at the nasal mucosae without requiring cellular uptake^61^. One strategy combines mucoadhesion with receptor mimicry to reinforce the mucus barrier and intercept virions before they reach the epithelium. Chitosan is a cationic polysaccharide with pronounced mucoadhesive properties arising from interactions with negatively charged mucins. Recently, a chitosan-sialic acid complex was shown to adhere to mucin and epithelial surfaces and to inhibit attachment and entry of influenza virus and respiratory syncytial virus. Intranasal administration of this formulation reduced respiratory viral infection and pulmonary inflammation in mice, providing direct proof of concept for a mucus-associated prophylactic strategy^62^.

Multivalent receptor mimics provide a complementary means of directly targeting influenza virions. Sialic-acid-functionalized gold nanoparticles, for example, bind multivalently to hemagglutinin (HA) and competitively inhibit influenza virus attachment to host cells^63^. Importantly, antiviral activity depends not only on ligand identity but also on how those ligands are presented. Linear polyglycerol-based polysialosides showed superior inhibition of influenza A virus compared with dendritic analogues and demonstrated efficacy both in vitro and in vivo in mice^26^. Multivalent inhibition is not restricted to carbohydrate ligands. Peptide-nanoparticle conjugates presenting multiple HA-binding peptides on dendritic polyglycerol scaffolds showed strong inhibition of influenza virus binding and infection in vitro and also demonstrated antiviral efficacy in vivo^64^. Spatial organization of receptor-mimicking ligands represents an additional design parameter; sialylated bacteriophage capsids with ligand spacing matched to the geometry of HA binding sites efficiently coated influenza virions and inhibited infection in vitro, ex vivo, and in vivo^25^. Multivalency can also be used to combine distinct mechanisms within a single construct. Heteromultivalent linear polyglycerols displaying both 6′-sialyllactose and zanamivir combine HA binding with neuraminidase inhibition and strongly suppress influenza A virus propagation in vitro and in human lung tissue ex vivo^65^.

An important distinction among extracellular antiviral materials is whether virus inhibition is reversible (virustatic) or irreversible (virucidal). Multivalent binding alone can prevent receptor engagement as long as the inhibitor remains associated with the virion, whereas appropriately designed macromolecules can additionally induce irreversible loss of infectivity. The virustatic principle has also been demonstrated with polymer-lipid nanomimics that mimic host-cell membrane components and reversibly sequester viral particles, thereby preventing cellular entry without inducing irreversible virion inactivation^66^. Sialylated β-cyclodextrins carrying 6′- or 3′-sialyllactosamine through long hydrophobic linkers showed broad anti-influenza activity and irreversible virucidal effects against human and avian influenza viruses, with activity confirmed across complementary experimental models^67^. Mechanistic studies subsequently showed that the 6′-sialyllactosamine moieties promote HA recognition, whereas the hydrophobic linker is required for interaction with the viral envelope and irreversible inactivation^68^. These findings illustrate how molecular architecture can determine not only antiviral potency, but also whether inhibition is transient or irreversible.

Local antiviral activity does not necessarily require virus-specific molecular recognition. Physicochemical approaches instead exploit vulnerabilities of the virion or its local environment. Acidified nasal formulations can exploit the pH sensitivity of influenza virus proteins and have been shown to rapidly inactivate influenza A viruses in vitro. In ferrets, low-pH nasal gel formulations reduced viral shedding and disease severity when administered early after challenge, demonstrating the potential of transient local acidification as a broad, non-target-specific antiviral strategy^69^. Nitric oxide-releasing nasal sprays represent another broad-spectrum approach. Recent studies demonstrated rapid virucidal activity of nitric oxide nasal spray formulations against influenza A and B viruses in vitro, although clinical efficacy has so far been established primarily in the context of SARS-CoV-2 rather than influenza^70^.

Together, these examples show that nasal antiviral strategies can act at several levels of the mucosal interface: by retaining or sequestering virions in mucus, preventing receptor engagement, irreversibly inactivating virus particles, or targeting infection after cellular entry. The relative value of these mechanisms depends not only on intrinsic antiviral potency, but also on local residence time, accessibility of the target, and distribution of the formulation within the nasal cavity. These route-specific requirements emphasize the need for experimental models that reproduce nasal epithelial physiology, mucus, and realistic formulation deposition.

The following sections therefore consider which animal and human-relevant experimental systems are best suited to address these different aspects of nasal antiviral development.

## Animal models for influenza virus infection and antiviral testing

For nasal antiviral development, the value of an animal model depends on the specific question being addressed. Relevant endpoints include antiviral efficacy in the upper respiratory tract, transmission, local deposition, pharmacokinetics, and systemic responses, and no single species reproduces all of these aspects equally well. Table 1 provides an overview of commonly used animal models, including their experimental readout parameters, virus strains, tested compounds, and translational applications. The models discussed below should therefore be considered complementary rather than as a simple hierarchy of increasing complexity.

**Table 1:** Overview of animal models used in influenza research

Model	Key readout parameters	Virus strains	Translational application	Tested compounds	References
**Mouse (BALB/c)**	Lung viral titers Mortality Body weight loss Lung inflammatory response Proinflammatory cytokines Pulmonary leukocyte infiltration	H1N1 H3N2 H5N1 H9N2 IBV	Antiviral efficacy; vaccine efficacy (lung-restricted readouts)	Amantadine EIDD2801 Oseltamivir Peramivir T-705 (favipiravir) Zanamivir	^78,189–191^
**Mouse (C57BL/6)**	Lung viral titers Mortality Body weight loss Lung inflammatory response Proinflammatory cytokines Pulmonary leukocyte infiltration	H1N1 H5N1 H7N9 IBV	Antiviral efficacy; immunopathology	Amantadine T-705 (favipiravir)	^78,192^
**Mouse (ICR)**	Lung viral titers Mortality Body weight loss Lung inflammatory response Proinflammatory cytokines Pulmonary leukocyte infiltration	H1N1	Antiviral efficacy	Baicalin Ribavirin	^193^
**Cotton rat**	Respiratory rate Fever Weight loss	H1N1 H3N2 IBV	Antiviral efficacy at the nasal mucosa (not suitable for transmission studies)	Oseltamivir Triamcinolone acetonide Zanamivir	^194,195^
**Guinea pig**	Mild clinical signs Nasal viral titers Seroconversion Transmission frequency to sentinel animals	H1N1 H3N2 H5Nx H7N9 IBV	Transmission studies; prophylactic/antiviral efficacy against onward transmission		^82,196^
**Golden Syrian hamster**	Weight loss No clinical signs High virus titers	H1N1 H2N2 H3N2 H5N1 H9N2 IBV	Transmission studies; vaccine efficacy		^71,197,198^
**Ferret**	Fever Weight loss Lethargy Viral titers Mortality Transmission	H1N1 H3N2 H5N1 IBV	Antiviral efficacy; pathogenesis; transmission	BXM Cyanovirin-N Cyclooctylamine hydrochloride EIDD2801 Fluororibosides Nitazoxanide Oseltamivir Peramivir Ribavirin Verdinexor Zanamivir + poly-L-glutamine	^190,199–207^
**Pig**	Nasal viral shedding Nasal swab viral titers Lung viral titers Clinical signs (fever, coughing, sneezing, nasal discharge) Lung pathology Cytokine responses Virus transmission	H1N1 (pdm09) H1N2 H3N2 H1N1 swine H3N2 swine	Antiviral efficacy; vaccine efficacy; transmission studies; regional nasal/lung drug deposition; pharmacokinetic evaluation of intranasal formulations	Oseltamivir Broadly neutralizing monoclonal antibodies (e.g., 2–12 C) Influenza vaccines	^89–91^
**Chicken**	Mortality Growth Egg production Clinical signs Viral titers	HPAI: H5Nx, H7Nx LPAI: H9N2	Vaccine efficacy; avian pathogenesis (not applicable to human influenza)	BXM (baloxavir marboxil) Oseltamivir Peramivir	^208,209^
**Non-human primate**	Viral load Body weight Proinflammatory cytokines	H1N1 H5N1 H5N6 H7N9	Pathogenesis/immunopathology studies	BXM Clarithromycin Peramivir Zanamivir	^210–212^
**Horse**	Clinical signs	H3N8 H7N7	Equine antiviral development (not applicable to human intranasal formulations)	BXM Peramivir	^95,96^

The table summarizes commonly used animal models, their principal experimental readouts, and their translational applications. Small-animal models are widely used for antiviral efficacy, pathogenesis, and transmission studies, whereas ferrets and pigs provide additional advantages for studies of respiratory physiology, transmission, drug deposition, and pharmacokinetics. Chickens and horses are primarily relevant to species-specific influenza research, and non-human primates are reserved for selected translational studies. Model selection should therefore be guided by the specific biological or pharmaceutical question.

Regarding the applied animal models, wild-type mice models, especially BALB/c and C57BL/6 mice, are often used. The advantages of these mice models in general are their cost-effectiveness, easy handling and their fast production rate, providing the ability to generate large numbers of data sets and therefore statistically robust data^71^. Mice develop measurable clinical and biological readouts, including body weight loss, survival rate, clinical score, lung weight index, viral titers in lung tissue (determined by plaque assay or TCID₅₀), and cytokine and chemokine profiles in lung homogenate or bronchoalveolar lavage fluid (BALF). Histopathological assessment of lung tissue by haematoxylin and eosin (H&E) staining, together with these parameters, can be used to evaluate the efficacy of antiviral substances and vaccines following intranasal infection^72^.

For nasal formulation testing, however, the distribution of the inoculum within the respiratory tract is a critical experimental variable. Restricting the intranasal inoculum to a small volume, typically administered to lightly sedated or unanaesthetised animals so that the liquid is retained in the nasal cavity by capillary action, confines infection largely to the upper respiratory tract (URT; nasal turbinates), providing a model that specifically mirrors the target site of intranasally delivered antivirals^73^. In contrast, larger intranasal volumes, particularly when administered under deep anaesthesia for intratracheal instillation, deposit virus into the trachea, bronchi and lungs and are used to model lower respiratory tract (LRT) disease and influenza pneumonia^73–75^. This volume-dependent distribution has been confirmed by imaging and radiotracer studies showing that the fraction of inoculum reaching the lungs increases with instilled volume, and the volume required to reach the lungs is species-dependent^73,74,76^. Selecting the appropriate volume and route of administration is therefore essential when using mice to evaluate nasal antiviral formulations, since an excessively large intranasal dose can shift the readout from an URT efficacy model toward LRT infection and pneumonia.

The main disadvantage of mouse models is their natural lack of susceptibility of the most inbred strains to the human influenza virus. Therefore, viral adaptation to the mouse species is required^71,72^. Most standard laboratory mouse strains carry defective Mx1 alleles and therefore lack an important component of innate resistance to influenza virus^77^. In contrast, *Mx1*-competent mice show stronger restriction of influenza virus replication and dissemination and can more closely reproduce aspects of the innate antiviral response present in natural hosts, although these mice are not standard laboratory strains and their use adds experimental complexity and cost^77^. In addition, mice do not efficiently recapitulate natural influenza virus transmission, particularly via respiratory droplets or aerosols, which limits their utility for transmission-focused studies. Furthermore, species-specific differences in innate and adaptive immune responses may complicate the direct translation of efficacy data for nasal formulations to the human virus-host interactions they are intended to model^78^.

In contrast to mice, cotton rats can be infected with influenza A and B viruses without prior viral adaptation. Viral replication occurs in both the upper and lower respiratory tract and is accompanied by pulmonary pathology and a robust cytokine response^78,79^. Cotton rats also develop measurable clinical signs, including increased respiratory rate and fever, providing useful readouts for antiviral efficacy studies^80^. However, unlike guinea pigs and ferrets, cotton rats are not established as a standard model for influenza transmission^80,81^. They are therefore most useful when local antiviral efficacy is the primary endpoint rather than onward transmission.

Transmission studies require a different set of model characteristics, Guinea pigs have become a well-established small-animal model for airborne influenza transmission, despite lacking the prominent coughing and sneezing seen in ferrets. Infected guinea pigs transmit virus to naïve cage-mates via passive aerosol shedding rather than active expulsion, and interventions can be evaluated by measuring nasal wash viral titers, seroconversion, and transmission frequency to sentinel animals^82,83^. This makes the guinea pig a particularly relevant model for testing whether a nasal antiviral spray reduces onward transmission, a key claim for prophylactic intranasal products. Guinea pigs show similar lung anatomy and physiology to humans and, in addition to their ease of handling and low costs, are naturally susceptible to human influenza viruses^82^. Golden Syrian hamsters provide another susceptible small-animal model and can support high levels of viral replication, but the relative paucity of overt clinical signs limits their use for studies in which disease severity is a major endpoint. They are therefore used mainly for transmission and vaccine studies^71,72^.

For questions that require both upper-airway infection and clinically recognizable disease, ferrets provide important advantages. Their respiratory anatomy and physiology closely resemble those of humans, and infected animals develop clinical signs including sneezing, coughing, nasal discharge, fever, and lethargy^84,85^. Infection with human influenza virus strains occurs predominantly in the upper respiratory tract^86^. Although ferrets remain less genetically tractable than mice, sequencing and annotation of the ferret genome have improved characterization of host responses to influenza infection, particularly through transcriptomic analyses and the interpretation of ferret-specific gene expression data^87^. Additionally, virus replication and transmission are similar to humans, leading to a promising model to study influenza virus pathogenesis, transmissibility, and the efficacy of active pharmaceutical ingredients (APIs)^84^. For ferrets, influenza serostatus is as important as specific pathogen-free (SPF) status when selecting animals for influenza studies. Because prior influenza exposure or maternal antibodies can alter disease outcomes and mask antiviral or vaccine efficacy. Therefore, the influenza serostatus of animals should be verified before inclusion in studies and confirmed seronegative to circulating influenza A and B viruses^88^. However, disadvantages of using ferrets as a model system for influenza is the limited accessibility of inbred or pathogen-free animals and the increased requirements for housing space, husbandry and handling^84^.

Where formulation delivery, device performance, or pharmacokinetics are central to the study, pigs offer advantages that smaller models cannot readily provide. Pigs are a large animal model of high physiological relevance for influenza infection and intranasal formulation testing. They are natural hosts of swine influenza A viruses and are also susceptible to infection with human-origin influenza A viruses. Following infection, pigs can develop clinical signs and pathological features resembling those observed in humans without requiring prior viral adaptation^89,90^. Their respiratory tract closely resembles that of humans in anatomy, physiology, and immunology, and their large nasal cavity accommodates clinically relevant delivery devices^91^. Moreover, intranasal, intratracheal, and aerosol delivery methods have been evaluated in pigs, making them a valuable model for studying regional drug deposition and the pharmacokinetics of intranasal antiviral formulations^91^. The main disadvantages of the pig model are the high cost, housing, husbandry and biocontainment requirements associated with a large animal species^89,90^.

Other species address more specialized questions and have limited relevance to routine nasal formulation development. Chickens are widely used for studies of avian influenza pathogenesis and vaccine efficacy but are not generally used to model human intranasal antiviral therapy^92^. Non-human primates (NHPs) share the closest relationship to humans in terms of genetics, immunological, anatomic and physiological similarities regarding respiratory infections^93^. Therefore, these models offer the best comparability with an infection in humans. However, the proximity to humans also entails ethical concerns and very high costs restricting their role to select confirmatory studies rather than routine formulation testing^94^. A rarer animal model in the context of influenza research are horses. Horses are primarily used for studies of equine influenza and are similarly of limited relevance to human nasal antiviral development^95,96^.

Each animal model offers different readout parameters, from cytokine release over various clinical symptoms up to mortality (Table 1). Although animal models that might be suitable for the investigation of influenza are available, the translation of animal data to the human situation remains as a challenge leading to a high translation failure rate. An ideal model for the translation of data should provide similar genetics, physiology and anatomy, disease pathophysiology, comparable endpoints to clinical studies and prediction of clinical efficacy. The careful evaluation and validation of animal models is crucial and remains as future challenge^94^. Furthermore, the ethical concerns using animal models are increasing and being addressed based on the 3 R principle (Replacement, Reduction and Refinement)^97^. These considerations are regulated in the European Directive 2010/63/EU^98^. Additionally, the Food and Drug Administration (FDA) Modernization Act 2.0 from December 2022 allows for alternatives to animal testing in preclinical studies^99^. In 2025, the FDA presented a roadmap to replace animal testing in preclinical drug safety studies with innovative New Approach Methodologies (NAMs). FDA pushes to replace animal testing^100^. This shift reflects several well-documented limitations of animal models for antiviral and biologic drug development. Interspecies differences in viral receptor tropism, immune signaling, and drug metabolism can limit the translatability of animal data to humans. In addition, in vivo studies with pathogenic viruses often require high-biocontainment facilities that constrain experimental throughput, and animal studies are costly, time-consuming, and raise ethical concerns^101–105^. Animal models are poor predictors of human safety for biologics and, more broadly, more than 90% of drugs that appear safe and effective in animals fail to gain FDA approval, predominantly because of safety or efficacy issues identified during human development^101^. Taking this into account, evaluated and validated in vitro or ex vivo alternatives are increasingly important for the investigation and screening of antiviral substances^101,103,106^.

In vitro and ex vivo platforms including human-derived cell lines, primary tissue explants, air-liquid-interface cultures, and organ-on-chip systems can overcome some of these constraints because they use human cells or tissues, are amenable to higher-throughput screening, and avoid interspecies variation^104–106^. However, these systems also have important limitations because they cannot fully reproduce the complexity of a living organism. Most in vitro and ex vivo systems lack a fully integrated innate and adaptive immune response, including immune-cell trafficking, humoral responses, and adaptive memory, and ex vivo cytokine responses have been shown not to reliably reflect the in vivo immune status^107,108^. They also cannot fully capture systemic pharmacokinetics, biodistribution, multi-organ crosstalk, or the longitudinal course of infection and pathogenesis in a whole organism^103,105,108^. Antiviral activity identified in a single cell type can also fail to generalize; for example, hydroxychloroquine potently inhibits SARS-CoV-2 in Vero E6 and Huh7 cells but is inactive in human Calu-3 lung cells, non-human primate and hamster infection models, and randomized clinical trials^104^. For these reasons, in vivo infection models remain necessary for questions that require an integrated organismal response and for confirming whether mechanistic findings and efficacy observed in vitro or ex vivo translate into a safe and effective antiviral response in an immunocompetent host^105,108^. In vitro, ex vivo and in vivo approaches therefore play complementary rather than interchangeable roles in antiviral drug development.

## Advanced experimental models for influenza virus infection and antiviral evaluation

A broad range of in vitro and ex vivo models has been established to study influenza virus infection, viral replication, and responses to antiviral treatment. These systems differ substantially in cellular complexity, physiological relevance, and experimental readouts, and therefore address different aspects of antiviral evaluation. Table 2 summarizes representative models and the corresponding readout parameters used across a range of influenza A virus subtypes, including H1N1, H3N2, H3N8, H5N1, H5N6, H7N7, H7N9, and H9N2, as well as additional viral systems used in comparative infection studies.

**Table 2:** Representative in vitro models used for influenza infection and antiviral evaluation

Experimental model/system	Readout parameters	Virus strains/variants	Tite/conditions	References
**MDCK cells**	Glutamate and aspartate increase as indicators of cell disruption and apoptosis	H3N8 (Newmarket 1/93, NIBSC)	Virus yield: 2.4 log HA/100 µL at 44 h post-transfection	^213^
**MDCK cells**	Adherent and detached cell concentrations Infection dynamics Virus-induced apoptosis and cell lysis Virus yields Total virus concentration (HA titration) Infectious virus concentration (TCID50 titration)	H1N1 (PR/8/34, NIBSC, Hertfordshire, UK) H1N1 (PR/8/34, Robert Koch Institute, Berlin, Germany) H3N2 (WSN/67/2005, NIBSC, Hertfordshire, UK)	2000 virus particles/cell for PR/8/34 NIBSC 20,000 virus particles/cell for PR/8/34 RKI 20,700 virus particles/cell for WSN/67/2005	^214^
**MDCK cells**	Antiviral efficacy based on virus yield (EC90): Baloxavir acid Oseltamivir acid Zanamivir hydrate Laninamivir Favipiravir	A/H1N1 (A/WSN/33) A/H1N1 (A/WSN/33-NA/H274Y) A/H1N1 (A/PR/8/34) A/H3N2 (A/Victoria/3/75) A/H3N2 (A/Hong Kong/8/68)	-	^215^
**MDCK cells**	Susceptibility testing in neuraminidase inhibition assays: Laninamivir Oseltamivir carboxylate Zanamivir Peramivir	A(H1N1) (WSN/33; Brisbane/59/07) A(H3N2) A(H1N1)pdm09	-	^216^
**MDCK cells**	H1N1 influenza A virus-induced apoptosis TEM analysis FCM assays	H1N1	-	^217^
**Human respiratory tissue and epithelial culture models** Ex vivo: conjunctiva, nasopharynx, bronchus, lung In vitro: nasopharyngeal, bronchial, and alveolar epithelial cells MDCK cells for virus replication	IFN-beta, IL-6, RANTES, and IP-10 protein expression Cytokine and chemokine gene/protein expression in NHBE cells Influenza A replication kinetics in respiratory epithelial cells Influenza matrix-gene expression	H1N1: A/Hong Kong/54/1998; A/Hong Kong/463721/2009; A/Hong Kong/415742/2009 H3N2: A/Oklahoma/1992/2005 H7N7: A/Netherland/33/2003 H5N1: A/Vietnam/3046/2004	-	^218^
**Human ex vivo respiratory tissues and primary cell cultures** Ex vivo: bronchus, lung, nasopharynx, tonsil In vitro: primary human alveolar epithelial cells and peripheral blood monocyte-derived macrophages	Virus tropism Induction of proinflammatory cytokine responses	H7N9: A/Shanghai/1/2013; A/Shanghai/2/2013 Highly pathogenic avian influenza H5N1 Highly pathogenic avian influenza H7N7 2009 pandemic H1N1 Low-pathogenic duck H7N9	-	^219^
**CEF cultures (chicken embryo fibroblasts)**	Astragalus polysaccharide (APS) concentrations Effect of APS on MHC and cytokine mRNA expression A570 values	H9N2 (AIV)	-	^220^
**Human ex vivo respiratory tissues and primary human alveolar epithelial cells (AECs)** Ex vivo: nasopharynx, bronchus, lung	Induction of IFN-beta, CCL2, CCL4, CCL5, and CXCL10	A/H5N6 H1N1pdm Avian H5N6/6154 H5N1/483 (control) H1N1pdm (control)	-	^221^
**RIM-MVECs**	TEM analysis HA-gene mRNA expression	H5N1	-	^222^
**MRC-5 cells and MDBK cells**	Trypsin dependence (plaque assay)	caMDV (human A/Hong Kong/97 [H5N1])	-	^223^

These in vitro models enable the investigation of early stages of the viral infection cascade, including viral entry and initial host responses, and therefore provide valuable alternatives to animal experimentation in early antiviral drug evaluation.

Selecting appropriate readouts is critical when evaluating a nasally administered antiviral formulation in vitro, since the relevant question is not only whether viral replication is reduced but whether the reduction reflects activity at the mucosal surface and is captured on a timescale relevant to topical (rather than systemic) drug exposure. Viral replication is most commonly quantified by infectious titers using tissue culture infectious dose (TCID_50_), hemagglutination (HA) assay, or plaque assay on MDCK cells, or by additionally quantitative reverse-transcription PCR (qRT-PCR), which measures genome copy number but does not distinguish infectious from non-infectious viral particles^109^. For nasal formulation testing, infectious-titers methods (TCID_50_, plaque assay) are reliable endpoint, while qRT-PCR remains useful as a rapid, high-throughput screening technique. Beyond quantitative virological endpoints, immunofluorescence (IF) microscopy and confocal imaging are routinely employed to provide cellular and spatial resolution of infection dynamics. Staining for influenza nucleoprotein (NP) or HA allows direct confirmation of productive infection at the single-cell level and enables quantification of infection rate as the percentage of antigen-positive cells. In differentiated epithelial models^110^, immunofluorescence and confocal microscopy can additionally be used to assess epithelial integrity and cell-type-specific infection by visualizing tight-junction proteins, ciliated and mucus-producing cells, and the spatial distribution of infected cells^110^. These readouts complement quantitative virological assays and allow treatment effects to be assessed through changes in viral antigen burden and preservation of epithelial architecture.

Conventional MDCK-based systems are widely used for influenza virus propagation and antiviral susceptibility testing^111^. Immortalized human airway epithelial cell lines, including A549, BEAS-2B, 16HBE14o-, and Calu-3, are also commonly used as two-dimensional models of influenza virus infection^112,113^. All of these cell lines have also been used under air-liquid interface (ALI) conditions, although their ability to establish a differentiated and stable epithelial barrier varies considerably. Calu-3 cells form a particularly robust polarized epithelial layer at ALI, whereas 16HBE14o- and BEAS-2B cells show more variable or limited polarization and barrier properties, and A549 cells lack the tight barrier phenotype characteristic of differentiated airway epithelium^42^. However, standard submerged cultures do not reproduce the differentiated cellular architecture and mucociliary phenotype of the native airway epithelium, and their transcriptional and innate immune responses can differ from those of differentiated primary airway epithelial cultures^42,114^. Consequently, antiviral activity observed in these simplified systems may not fully predict activity in differentiated human airway tissue^42^.

In contrast to models derived from lower-airway tissues, primary nasal epithelial cells cultured at an air-liquid interface (ALI) provide a physiologically relevant model of nasal mucosa. These cells are typically obtained by nasal brushing or biopsy and cultured on permeable supports under ALI conditions, where they differentiate into a pseudostratified epithelium containing ciliated cells, mucus-producing goblet cells, and basal cells^115^. Depending on the donor and differentiation protocol, primary airway ALI cultures can also contain less abundant epithelial populations, including club or other secretory cells, ionocytes, neuroendocrine cells, and deuterosomal precursor cells involved in multiciliated-cell differentiation.

Differentiated primary nasal epithelial cultures support productive influenza A virus infection and can therefore be used to investigate viral replication, epithelial responses, and antiviral interventions directly at the nasal mucosal surface. Peretz et al. demonstrated influenza A virus infection and antiviral responses in differentiated primary human nasal epithelial cells^116^, whereas Richard et al. showed efficient infection of fully differentiated primary human nasal respiratory epithelial cultures by human H1N1 and H3N2 viruses^13^. In a standardized 3D nasal ALI model, oseltamivir reduced influenza A virus replication and preserved epithelial barrier function and mucociliary activity^13,116,117^.

Immortalized nasal epithelial cell lines provide a complementary but less physiologically differentiated model. RPMI 2650 cells, which originate from a human nasal squamous cell carcinoma, can form a polarized epithelial barrier under ALI conditions and are widely used for permeability and nasal drug-transport studies. However, they do not reproduce the fully differentiated ciliated pseudostratified respiratory epithelium generated by primary nasal epithelial cells and should therefore not be considered equivalent to primary nasal ALI cultures^118,119^.

Primary human airway epithelial cells derived from bronchial or other lower-airway tissues are also frequently cultured at ALI and differentiate into pseudostratified respiratory epithelia containing basal, ciliated, mucus-producing, and other secretory cell populations^120^. These models are highly valuable for studying influenza infection and lower-airway responses, but their anatomical origin should be considered when extrapolating findings to intranasally applied formulations. Commercial ALI systems based on primary bronchial or nasal cells are available from several suppliers; however, donor-specific characteristics can contribute to inter-donor variability in epithelial morphology and experimental responses. The use of single-donor versus pooled-donor cultures therefore represents an important experimental-design consideration, as pooling may reduce the impact of responses specific to individual donors^121,122^.

Beyond differentiated epithelial ALI cultures, co-culture and triple-culture systems have been developed to incorporate additional cell types and thereby capture aspects of intercellular signaling that are absent from epithelial monocultures. These models combine airway epithelial cells with immune or stromal cells, such as monocyte-derived dendritic cells and fibroblasts, and can better reproduce proinflammatory cytokine responses to influenza infection^123^. ARLO cells, a recently established monoclonal human alveolar epithelial line derived from immortalized primary cells rather than cancerous tissue, provide an additional epithelial model with improved barrier properties compared with conventional tumor-derived cell lines^124^.

A key limitation of the standardized ALI system is difficulty in scaling up, since cells are grown on a defined, membrane area that may not be representative of the in vivo tissue. Organoid and spheroid models were developed to address this^125^. Human nose and lung organoids, grown in an extracellular matrix with a nutrient gradient, recapitulate airway tissue and reveal cell-type-specific infectivity and epithelial damage by respiratory viruses including influenza, rhinovirus, RSV, and SARS-CoV-2^125–127^. These models can capture variant-dependent infectivity and age-dependent susceptibility, and are particularly useful for viruses that are otherwise difficult to cultivate, such as zoonotic strains^128^. Airway organoids are widely used to model host-pathogen interactions given their cellular composition, which closely resembles the human airway epithelium^129^. However, their basal-out polarity with the apical surface facing an inaccessible internal lumen restricts access to the epithelial surface most relevant to infection and drug application, complicating host-pathogen interaction studies^125^. Strategies to overcome this include microinjection into the organoid lumen^130^. This configuration enables direct infection with respiratory viruses including influenza A virus and SARS-CoV-2 without the need for microinjection or mechanical disruption^125^.

Despite these advantages, the longer differentiation time and higher cost of maintaining induced pluripotent stem cell-derived organoids currently limit routine use. Nevertheless, ALI and organoid models provide complementary platforms for studying influenza infection. Ekanger et al. compared ALI cultures and airway organoid models for influenza A/H1N1 and found similar viral replication kinetics, cytokine release, and serum-mediated neutralization despite differences in cell composition and differentiation, indicating that both platforms are valuable depending on the specific research question^127^.

Precision-cut lung slices (PCLS) preserve native lung architecture and cellular complexity, including resident epithelial and immune cell populations, and therefore provide a physiologically relevant ex vivo model of the lower respiratory tract^131^. Human PCLS support productive infection with influenza A viruses, including H1N1 and H5N1, and reproduce virus-induced antiviral and inflammatory responses, including interferon and cytokine induction^132^. Their multicellular organization enables investigation of viral tropism and tissue-level host responses that cannot be fully reproduced in epithelial monocultures. Comparisons with primary type II alveolar epithelial cells (AECII) and A549 cells further illustrate the influence of model complexity: A549 cells generally show a more limited cytokine response, although induction of individual interferon-stimulated genes can be comparable or even increased, indicating gene-specific rather than uniformly reduced antiviral responses in immortalized cells^133^. Complementary ex vivo human lung tissue studies have identified type II pneumocytes as major targets of influenza A virus in the lower respiratory tract and showed that a novel H7N9 strain, unlike avian H7 viruses, replicated efficiently because of adaptive mutations and NS1-mediated suppression of the antiviral interferon response^134^. Together, these tissue-based systems provide valuable platforms for investigating influenza virus replication, tropism, and host responses in the human lower respiratory tract. However, PCLS are limited by relatively short-term viability, donor-to-donor variability, and sensitivity to tissue handling and preparation, which can affect experimental reproducibility^131^.

For preclinical evaluation of antiviral nasal products, ex vivo models derived directly from nasal tissue provide greater anatomical relevance to the upper airway mucosa. Human inferior nasal turbinate tissues maintained as organ cultures preserve the multicellular architecture of the nasal mucosa and support productive SARS-CoV-2 infection, enabling analysis of viral tropism and local innate immune responses^135^. Similarly, porcine nasal mucosal explants cultured at an air-liquid interface support influenza A virus infection and reproduce key antiviral gene-expression responses observed in respiratory tissues in vivo^136^.

Microfluidic organ-on-chip technologies have more recently been developed to model lung physiology, inflammation, and viral infection within controlled microenvironments, integrating epithelial and endothelial compartments within microfluidic devices to reproduce key aspects of tissue-level physiology and vascular perfusion^137^. Lung-on-chip platforms allow improved control of the cellular microenvironment and can support antiviral screening under more physiologically relevant conditions^138^. Applications specifically modeling influenza infection include a bronchial airway-on-chip lined by differentiated human airway epithelium and pulmonary endothelium, which recapitulated virus infection, and cytokine production, and was used to show that co-administration of nafamostat with oseltamivir doubled the treatment-time window for oseltamivir in influenza A-infected chips^137,138^. The airway-chip platform has additionally been used to model clinically relevant influenza virus evolution and the emergence of antiviral resistance^138^.

3D bioprinting is a further emerging approach, in which cells and biomaterials are printed in defined spatial arrangements to generate structured tissue models for antiviral evaluation^139^. De Santis et al. established a 3D airway model using a bioink of decellularized extracellular matrix proteins combined with alginate, smooth muscle cells, and primary airway epithelial cells^140^. Berg et al. developed a bioprinted lung model of primary lung fibroblasts and THP-1 cells in a hydrogel matrix with an A549 epithelial layer printed on top, in which oseltamivir treatment produced a dose-dependent reduction in influenza A replication^141^, and subsequently extended this to a four-cell-type ALI-cultured model infected with both influenza A and SARS-CoV-2^142^. A very recent study created 3D alveolar epithelial sacculoids by bioprinting pluripotent stem cell-derived alveolar epithelial type II cells into defined high-density geometries^143^. The cells self-organized into lumenized sacs and exhibited a high degree of physiological functionality. Ventilation of these sacculoids induced volumetric oscillations, and the model closely recapitulated key physiological features of the human alveolus, including physiologically relevant host responses following apical infection with influenza A virus. Although the application of this model to nasal spray formulations has not yet been demonstrated, its high physiological relevance makes it a promising platform for investigating respiratory virus infections and evaluating novel inhaled antiviral therapeutics. Supporting this perspective, a recent study demonstrated that a jet nebulizer can efficiently deliver siRNAs to THP-1 macrophage-like cells in vitro^144^. The combination of advanced bioprinted respiratory models with aerosol or nebulization technologies may therefore provide a valuable platform for the preclinical evaluation of novel antiviral delivery formulations under physiologically relevant conditions. The major advantage of 3D bioprinting is its high degree of tissue specificity and structural control, though biomaterials require purification to remove residual immunogenic components that could otherwise trigger confounding inflammatory responses^145^.

Despite these challenges, advanced bioprinted respiratory models are expected to become increasingly valuable tools for influenza research and may also support the preclinical development of novel inhaled antiviral therapies, including nasal spray formulations. Table 3 provides an overview of three-dimensional culture models for studying influenza virus infection.

**Table 3:** Overview of three-dimensional culture models for studying influenza virus infection

Model	Readout parameters	Virus strain(s)	Titer/conditions	References
Human airway organoids (3D)	Viral replication kinetics (qRT-PCR, infectious titer); cell tropism (immunostaining)	Influenza A (H7N9, H1N1pdm, H3N2)	Organoid infection at MOI typically 0.01–1; progressive virus replication over 48–96 h	^129^
3D airway epithelial cultures (ALI)	Virus infection and spread; epithelial integrity	Influenza A (PR/8/34, H1N1)	Apical infection of differentiated epithelium under ALI conditions	^224^
Human precision-cut lung slices (PCLS)	Viral replication (plaque/TCID₅₀); cell tropism	Influenza A (H1N1, H3N2; strain-dependent)	Infection at defined inoculum (10⁵ PFU per ml); maintained 24–72 h ex vivo	^225^
Immune-competent lung-on-a-chip	Viral replication; barrier disruption; cytokine/chemokine signaling; immune cell recruitment and activation	Influenza A (H1N1)	Apical infection at MOI = 0.1-10; ALI with circulating immune cells; analysis up to 72 h	^226^
Human lung alveolus chip (organ-on-chip, ALI)	Viral replication (plaque assay, RT-qPCR); inflammatory response (IL-6, MCP-1, RANTES); immune cell recruitment; off-target effects (RNA-seq)	Influenza A (H1N1, H3N2)	Apical infection at MOI = 1; Cas13d–crRNA treatment 5 h post-infection; dynamic flow and breathing motions; analysis up to 72 h	^227^
Bioprinted four-cell-type human lung model (ALI, 3D)	Viral replication (plaque assay, qRT-PCR); epithelial susceptibility; immune response; cytokine release (IL-29); cell viability; tissue architecture (immunostaining)	Influenza A (H3N2)	Apical infection at MOI = 0.01; analysis over 24–72 h in bioprinted constructs	^142^
3D airway epithelial cultures (ALI)	Viral replication (plaque assay, qPCR); epithelial morphology and integrity; cell tropism; transcriptional and protein immune responses (IFNB1, IFNL1, IL-6, IL-8); viral entry factor expression	Influenza A H1N1	Apical infection at MOI = 0.001; monitored 1–9 dpi; viral titers from apical washes; gene expression by qPCR; protein secretion by ELISA/protein array; morphology by H&E and immunofluorescence	^228^
Human nasal epithelial cells (hNECs, ALI)	Viral replication kinetics (plaque assay); cell tropism (immunofluorescence: ciliated & goblet cells); host response (RNA-seq DEGs, qPCR); cytokine/chemokine secretion (Luminex: IFNβ, IL-1α/β, CXCL10); antiviral drug response (peruvoside)	Seasonal influenza A (H1N1, H3N2), Influenza B (Victoria lineage), Avian influenza A (H5N1)	Infection at MOI 0.1 (all viruses) and MOI 1.0 (H5N1); timepoints at 8, 24, 48 hpi; H5N1 shows lower replication (10⁵–10⁶ PFU/mL vs 10⁷–10⁸ for seasonal)	^229^
Ex vivo human lung tissue + primary alveolar macrophages + AEC II	Interferon response (IFN-α2, IFN-β, IFN-γ, IFN-λ1); cytokine production (IL-1β, GM-CSF, IL-6, IL-8, IL-10, TNF-α); IL-1β–GM-CSF axis; viral localization (immunostaining); IL-1β mRNA and protein expression; GM-CSF induction by IL-1β; effect of IFN on cytokine suppression; bacterial growth; pharmacological rescue via Tyk2 inhibition	Influenza A (Pan/99 H3N2),	IAV infection: 1×10⁶ PFU for 24 h; IFN stimulation (β, γ, λ1: 100 U/mL); IL-1β stimulation (5–10 ng/mL); TNF-α (100 U/mL); IL-1 receptor antagonist (anakinra: 1 µg/mL); Tyk2 inhibitor (PRT2070: 1 µM)	^230^

The table summarizes commonly used 3D in vitro models, including organoids, spheroids, and advanced airway models, together with their main applications for investigating host–pathogen interactions and evaluating antiviral therapies.

Importantly, many of the more complex respiratory models discussed above, including PCLS, lung-on-chip platforms, and bioprinted tissues, represent bronchial or alveolar rather than nasal compartments. Their findings should therefore be extrapolated to intranasal antiviral therapy with caution. Further development of nasal-specific organoid, microphysiological, and deposition-integrated models will be important for improving the translational relevance of preclinical nasal antiviral testing.

## Opportunities for alternative models to animal testing in the development of antiviral nasal formulations

For antiviral nasal formulations, model selection must consider not only biological relevance but also compatibility with the intended route of administration. Nasal sprays interact directly with mucus and the apical epithelial surface, while their distribution is additionally shaped by spray characteristics and nasal airflow. Experimental systems therefore differ substantially in their ability to reproduce formulation exposure and deposition, making the mode of administration a key criterion for selecting an appropriate model.

**Fig. 2:**
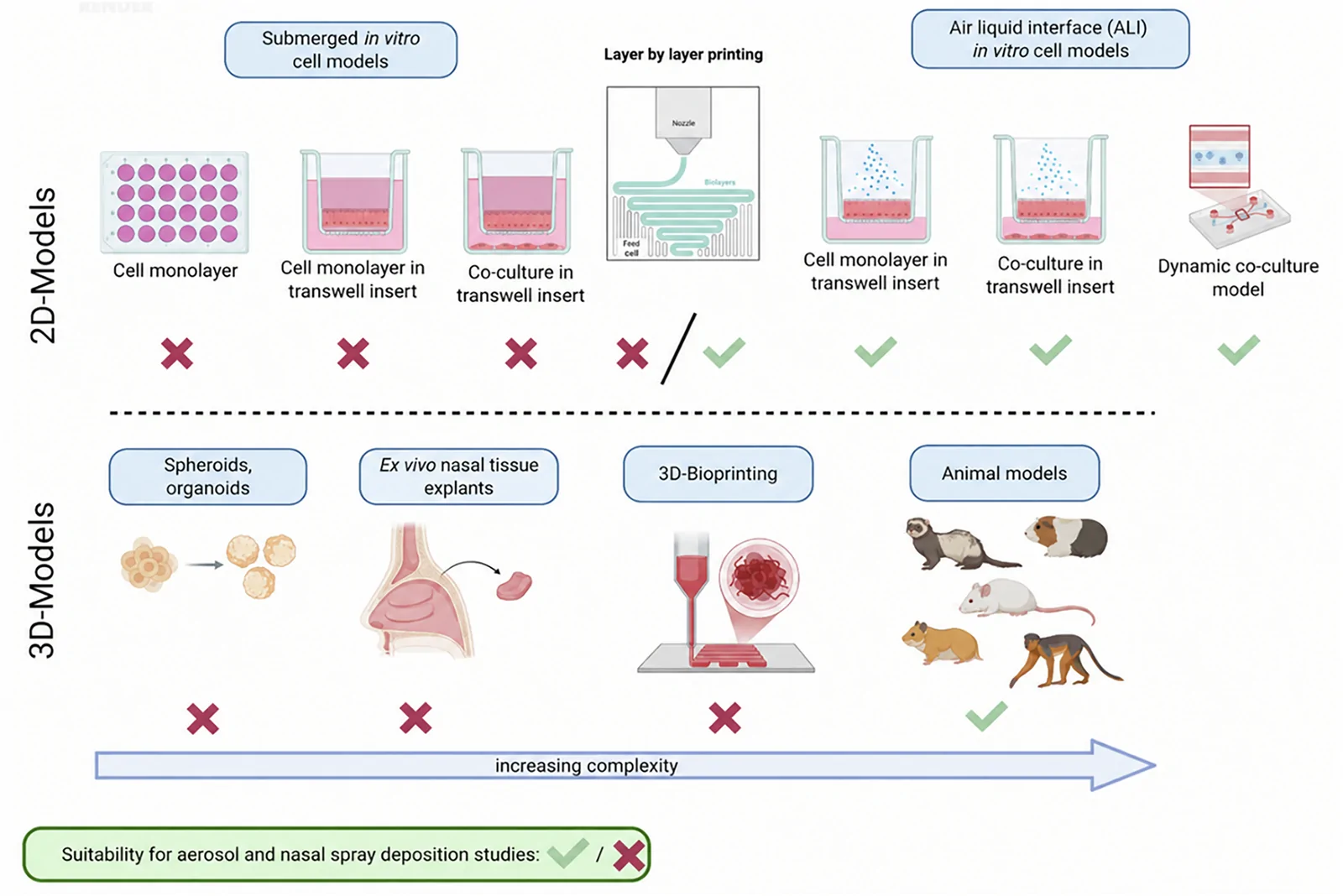
Overview of experimental models and their suitability for aerosol and nasal spray deposition studies. Experimental platforms are arranged according to increasing model complexity and include conventional submerged 2D cultures, layer-by-layer printed systems, air-liquid interface (ALI) cultures, dynamic co-culture models, spheroids and organoids, ex vivo nasal tissue explants, 3D-bioprinted models, and animal models. Submerged cultures, spheroids/organoids, ex vivo nasal tissue explants, and most 3D-bioprinted systems are valuable for infection, toxicity, and mechanistic studies but generally provide limited suitability for direct aerosol or nasal spray deposition. In contrast, ALI cultures and selected dynamic or engineered exposure systems provide direct access to the apical epithelial surface and are therefore better suited for controlled aerosol application. Animal models enable whole-organism assessment of deposition and therapeutic responses but are associated with species-specific differences and ethical considerations. The figure highlights that model selection should depend on the biological question and the intended mode of administration rather than on model complexity alone. This image was created with BioRender.com.

Figure 2 summarizes the experimental models discussed above and compares their suitability for nasal spray and aerosol exposure studies. Importantly, because these platforms differ in the accessibility of their apical epithelial surface therefore, each requires a distinct strategy for administering nasal sprays, aerosols or formulations, as they cannot be applied identically across submerged cultures, ALI systems, organoids/spheroids, and organ-on-chip devices, and the administration approach relevant to each platform is described below. Conventional submerged cell culture models remain the most widely used systems for antiviral screening and basic infection studies due to their simplicity, low cost, and high throughput. However, because these cultures are completely covered by liquid, the epithelial surface is not exposed to air. This prevents realistic aerosol contact with the cells and therefore limits their applicability for studying nasal spray delivery^146^. In these systems, test formulations are typically introduced as a diluted liquid added directly to the apical culture medium rather than delivered as a discrete spray plume or aerosol cloud, so the droplet size distribution, spray velocity, and deposition pattern characteristic of an actual nasal spray device cannot be reproduced^147^.

In contrast, nasal epithelial cells cultured at air-liquid interface (ALI) expose the apical epithelial surface directly to air and therefore allow controlled aerosol contact with the epithelium. Differentiated ALI cultures reproduce key structural and functional features of airway epithelium, including mucus production and ciliary activity, and therefore represent one of the most suitable in vitro systems for studying antiviral nasal formulations. Administration to ALI cultures is achieved by applying the spray or aerosol directly onto the exposed apical surface using a nebulizer, spray device, or dedicated aerosol exposure chamber, allowing the dose, droplet size, and deposition pattern of the test formulation to more closely approximate in vivo nasal spray administration^148,149^.

Organoid and spheroid models offer a three-dimensional microenvironment that reproduces important aspects of epithelial tissue organization and host-pathogen interactions. These systems can provide valuable insights into viral infection mechanisms and cellular responses. However, their architecture often restricts access to the apical epithelial surface, which limits the feasibility of aerosol exposure experiments and reduces their suitability for evaluating nasal spray delivery^150–152^. This constraint arises directly from the administration route because the apical epithelial surface of a conventional organoid faces an internal, fluid-filled lumen. Hence, spray or aerosol formulations cannot be applied externally and must instead be delivered via intraluminal microinjection or by mechanically disrupting the organoid architecture, thereby compromising tissue integrity. Newer apical-out organoid configurations, in which epithelial polarity is inverted so that the apical surface faces outward, allow more direct aerosol application and are beginning to address this administration barrier, although they remain less established for nasal spray testing than ALI cultures^125,130^.

Organ-on-a-chip platforms represent a further development of in vitro modeling technologies by incorporating microfluidic channels that allow dynamic flow conditions, mechanical stimulation, and controlled microenvironments. These systems can reproduce aspects of airway physiology such as airflow and tissue-vascular interactions and may therefore offer promising opportunities for studying respiratory infections and drug delivery. Nevertheless, their technical complexity and the current lack of standardized device designs and experimental protocols can limit reproducibility and broader application^153^. Administration is difficult for these platforms as most current nasal and airway organ-on-chip designs deliver test compounds as solutions or suspensions perfused through the device’s microfluidic channels rather than as true aerosols, and therefore do not reproduce the dosimetry of an actual nasal spray. However, microfluidic nasal models have also been adapted for the delivery of aerosolized formulations, enabling analysis of aerosol deposition, epithelial barrier responses, and drug transport under controlled flow conditions^147,154^. Only a limited number of specialized chip designs incorporate a dedicated air channel that allows aerosolized formulations to be deposited directly onto an ALI-cultured epithelium under controlled, adjustable airflow, and such aerosol-capable chips remain technically demanding to fabricate and are not yet widely standardized^148,149^.

Ex vivo respiratory tissue models preserve native tissue architecture and cellular composition, but their suitability for nasal spray testing is limited by the way they are typically cultured and exposed. Many tissue explants and slices are maintained under submerged conditions, which restricts direct access of spray or aerosol formulations to the epithelial surface. They are therefore better suited to short-term infection, toxicity, and mechanistic studies than to realistic assessment of nasal spray deposition or aerosol exposure^105^.

Engineered tissue models generated by layer-by-layer printing represent a promising strategy for constructing structured tissues with improved spatial control and could complement existing 3D and microengineered systems for aerosol drug delivery^155^.

## Nasal spray devices, aerosol exposure, and deposition models

To investigate nasal drug delivery systems under controlled conditions, several aerosol exposure platforms have been developed. Various in vitro and ex vivo systems originally designed for pulmonary drug delivery have been summarized by Primavessy et al., demonstrating the wide range of experimental tools that may also be adapted for nasal administration studies^156^.

Automated bioaerosol exposure systems using collision nebulizers can generate virus-containing aerosols that are delivered into exposure chambers under controlled temperature and humidity conditions^157^. In such setups, epithelial cells cultured under ALI conditions can be positioned within the exposure chamber, allowing controlled exposure to aerosolized virus particles. The concentration of aerosolized virus can be quantified using biosamplers and spray factor calculations. In a recent study, a commercial jet nebulizer was used to deliver small interfering RNAs (siRNAs) in combination with a delivery agent. This setup provides a platform for optimizing siRNA-containing aerosol formulations in vitro^144^.

Such exposure systems can provide controlled delivery of aerosolized compounds to compatible in vitro models, particularly ALI cultures. However, their relevance to nasal spray development depends on whether the delivered dose, droplet size, deposition pattern, and epithelial exposure adequately represent the intended clinical route. They should therefore be regarded as model-specific exposure tools rather than as validated surrogates of clinical nasal spray performance. Clinically used inhaled antivirals provide examples of device-based local respiratory delivery, although these approaches should be distinguished from nasal spray administration. Zanamivir has been administered as a dry powder by oral inhalation using the Diskhaler device for the treatment of influenza, whereas aerosolized ribavirin has been evaluated by nebulization in patients with influenza^158,159^. These examples demonstrate the feasibility of local respiratory antiviral delivery but primarily target the respiratory tract rather than the nasal mucosa.

In addition, 3D-printed nasal casts represent valuable tools for studying regional nasal drug deposition and the influence of anatomical variability on formulation performance^160,161^. When combined with computational fluid dynamics (CFD), these models can also be used to investigate airflow patterns and aerosol transport within the nasal cavity^162^.

Importantly, because nasal anatomy varies considerably between individuals, several studies have used 3D-printed nasal casts derived from multiple CT-based geometries to directly capture this inter-individual variability and its impact on drug deposition. Rigaut et al. fabricated nasal replicas from 11 different CT scans and showed that specific anatomical features, such as the nasal valve area and olfactory region geometry, significantly influenced regional powder deposition in the olfactory region, informing device and administration parameters for personalized nose-to-brain delivery^160^.

Similarly, Pasteur et al. characterized nasal cavity anatomy from 98 computed tomography scans in a European patient population, identified distinct anatomical subgroups, and 3D-printed representative nasal casts for each group, demonstrating that anatomical variation meaningfully altered deposition profiles depending on the delivery device used^161^. Together, these studies illustrate that 3D-printed nasal casts can be used not only to model an idealized average nasal geometry, but also to generate patient-specific or population-representative deposition data relevant to nasal formulation and device development.

Maaz et al. developed an ALI-cultured RPMI 2650 nasal epithelial model integrated into a three-dimensional human nasal cast to evaluate the deposition, biocompatibility, and transepithelial transport of aerosolized PLGA nanoparticles delivered via a pressurized metered-dose inhaler. This study demonstrated that the ALI model maintained cell viability and barrier function while enabling realistic assessment of nanoparticle deposition and transport, highlighting its value as a useful preclinical screening platform for intranasal drug delivery systems^163^.

Furthermore, 3D-printed nasal anatomical models can be directly combined with computational fluid dynamics (CFD) simulations to create in silico-in vitro hybrid approaches that predict airflow patterns and aerosol deposition in individual patient anatomies^162^. This integrated platform enables formulation developers to optimize spray characteristics, predict in vivo performance, and identify optimal delivery conditions before conducting resource-intensive and ethically challenging animal studies. The ability to incorporate anatomical variation also enables assessment of formulation robustness across different nasal cavity geometries.

The Alberta Idealized Nasal Inlet (AINI) represents a validated in vitro model that replicates complex nasal anatomy, enabling quantification of drug regional deposition across four distinct zones i.e., vestibule, turbinates, olfactory region, and nasopharynx^164^. The AINI has demonstrated strong in vitro-in vivo correlations (IVIVC) for nasal spray deposition, permitting differentiation between formulations and devices based on regional particle deposition^164^. More recently, the model has also been applied to characterize intranasally delivered dry powders^165^.

The most promising candidates can then proceed to confirmatory animal studies which remain important for investigating whole-organism aspects of viral infection and therapeutic intervention that cannot yet be adequately reproduced in vitro. However, interspecies differences in respiratory anatomy, immune responses, and nasal airflow patterns can limit their predictive value for human nasal drug delivery. Ethical considerations and regulatory efforts to implement the principles of replacement, reduction, and refinement further highlight the need for alternative experimental strategies. Rather than treating the models above as independent, interchangeable options, they can be organized into a stepwise, tiered translational framework in which in vitro, ex vivo, and in vivo approaches are used in a complementary and sequential manner. Each tier informs decisions about whether a candidate formulation advances to the next stage. In Tier 1 (early screening), conventional submerged epithelial cultures and organoid/spheroid systems are used for high-throughput evaluation of antiviral potency, cytotoxicity, and host-pathogen interaction mechanisms, allowing rapid low-cost triage of candidate formulations before more resource-intensive testing.

In Tier 2 (functional and aerosol delivery characterization), differentiated ALI cultures and organ-on-a-chip platforms, coupled with bioaerosol exposure systems, are used to evaluate antiviral efficacy under conditions that reproduce mucus production, ciliary activity, and airflow-dependent aerosol deposition, bridging the gap between simple monolayer assays and whole-tissue physiology.

In Tier 3 (anatomical and biopharmaceutical characterization), ex vivo nasal tissue slices, 3D-printed nasal casts, and the AINI model, combined with CFD simulations, are used to characterize regional drug deposition, transepithelial transport, and formulation robustness across realistic and anatomically variable nasal geometries, allowing spray and device parameters to be optimized before whole-organism testing.

Only candidates that demonstrate consistent antiviral efficacy, favorable regional deposition, and acceptable biocompatibility across Tiers 1–3 would then proceed to Tier 4, confirmatory in vivo testing, which remains necessary to capture systemic pharmacokinetics, innate and adaptive immune responses, and whole-organism toxicity that cannot yet be fully reproduced in vitro^166,167^. This tiered structure mirrors strategies already applied in inhalation toxicology and safety pharmacology, where models of progressively increasing complexity are used to preserve human translatability while reducing animal use^168^, and it directly supports the 3Rs principles of replacement, reduction, and refinement by reserving animal experiments for late-stage confirmation of the most promising candidates rather than for early-stage screening^169^.

Together, these experimental platforms illustrate how combinations of advanced in vitro models and aerosol delivery systems can support the development and evaluation of antiviral nasal formulations. Rather than serving as direct replacements for animal models, these human-relevant systems represent complementary tools that can improve the prediction of human responses during early stages of pharmaceutical development. Integrating advanced in vitro models with targeted in vivo studies within a stepwise translational framework will facilitate the validation of NAMs, further improve their physiological relevance, and ultimately support a gradual transition towards more human-based approaches while reducing the reliance on animal testing.

## Regulatory considerations for the development of antiviral nasal spray formulations

The development of new nasal spray formulations requires compliance with several regulatory guidelines and quality standards. Compared with systemically administered antivirals, intranasal products require additional route- and device-specific assessment, including local tolerability, nasal deposition, spray performance and potential systemic exposure^170,171^. For example, the development of the intranasal live attenuated influenza vaccine FluMist/Fluenz illustrates these requirements, as its assessment included nasal deposition and distribution as well as local and systemic immune responses^172,173^. However, it also illustrates current limitations of new approach methodologies (NAMs): although human airway and deposition models can support the assessment of local delivery, viral replication and epithelial responses, they cannot yet fully reproduce the whole immune response. Regulatory classification is primarily determined by the intended purpose and principal mode of action rather than by whether an effect is described as virucidal, virustatic or direct-acting antiviral. Products acting pharmacologically, immunologically or metabolically are generally regulated as medicinal products, whereas products acting primarily through a physical barrier or mechanical virus trapping may qualify as medical devices, subject to case-by-case assessment^174,175^. For development in the United States, the CDC/ACIP recommendations guidance on drugs for influenza treatment and prophylaxis should additionally be considered^176^.

Currently, the requirements for the approval of active pharmaceutical ingredient (API)-containing nasal sprays are primarily defined by regulatory guidance issued by European and United States authorities. The EMA Guideline on the Pharmaceutical Quality of Inhalation and Nasal Products came into effect in 2006^177^. In the United States, the Food and Drug Administration (FDA) provides regulatory guidance for nasal spray and inhalation drug products, particularly regarding chemistry, manufacturing, and controls testing requirements^178^. Pharmaceutical development must be considered within the framework of International Council for Harmonisation (ICH) guidelines governing pharmaceutical development and manufacturing^179^. For the development of generic or biosimilar nasal formulations, regulatory agencies require demonstration of bioequivalence with already approved products. Bioequivalence assessment of locally acting nasal spray and nasal aerosol products may incorporate evidence from in vitro performance, systemic exposure, and local drug delivery, together with appropriate formulation and device considerations^180^. Importantly, the United States Code of Federal Regulations (CFR §320.24) specifies that bioequivalence may be demonstrated through human in vivo studies or validated in vitro tests that are predictive of human bioavailability. Notably, this regulation does not explicitly require animal models for demonstrating bioequivalence.

Several reviews have summarized the development pathways for nasal drug products and inhalation therapies. For example, Scherließ and Trows et al. provide comprehensive overviews of formulation development, device characterization, and regulatory requirements for nasal drug delivery systems^181,182^. Further perspectives on in vitro testing approaches for orally inhaled drug products have been discussed by Forbes et al.^183^. These analyses highlight that many regulatory requirements for device and formulation characterization rely predominantly on in vitro testing methods, including particle size measurements, spray pattern analysis, plume geometry evaluation, and aerodynamic characterization. The extensive use of such methods underlines the predictive value of in vitro tools for the development and quality control of nasal drug products.

For safety-related questions, however, animal studies are still commonly included during the preclinical phase of drug development^184^. Toxicological considerations for drugs administered via non-conventional routes, including nasal administration, have been reviewed in detail by Emami et al.^185^. The regulatory landscape is nevertheless evolving. The FDA Modernization Act 2.0 broadened the types of nonclinical evidence that may be considered in drug development and facilitated the use of alternatives to conventional animal testing^99^. In 2025, the U.S. Food and Drug Administration published a strategic roadmap to reduce reliance on animal testing in preclinical safety studies through greater integration of New Approach Methodologies (NAMs), including organ-on-chip systems, organoids, and computational approaches^101^. In 2026, the FDA reported progress toward the first-year goals of this roadmap, indicating continued implementation of this transition toward more human-relevant nonclinical testing strategies^103^.

Despite this progress, NAMs cannot yet universally replace animal models, and their suitability depends on the specific context of use^186,187^. Importantly, increased physiological complexity or human origin of a model does not by itself establish predictive validity; this must be demonstrated for a defined context of use by comparison with relevant animal or clinical data^186,187^. Consequently, NAM-derived data may still need to be complemented by animal or clinical evidence until sufficient validation has been established for the intended application^186,187^.

Also, identifying conditions such as drug targeting human-specific receptors where animal studies can be excluded by utilizing in vitro and computational models^186^. With regard to the specific evaluation of antiviral nasal sprays targeting influenza virus infection, there is currently no dedicated regulatory guidance describing standardized in vitro efficacy models^187^. “Future advanced testing strategies have to combine advanced in vitro infection models with regulatory principles such as Good In Vitro Method Practices (GIVIMP). To be acceptable from EMA or FDA, new alternative methods require validation according to the GIVIMP or the recently published FDA Guidance draft on “General Considerations for the Use of New Approach Methodologies in Drug Development”^188^. To simulate nasal administration during preclinical development, several experimental platforms are already available, including in vitro aerosol exposure systems, nasal deposition models, and anatomical nasal casts. These systems provide valuable opportunities to investigate nasal drug delivery and antiviral efficacy under controlled laboratory conditions.

## Conclusion

Nasal sprays represent a versatile platform for the delivery of antiviral agents, offering several advantages including targeted local therapy at the nasal mucosa during the early phase of infection, rapid and convenient administration, and improved patient acceptance. Antiviral strategies explored for nasal delivery have primarily focused on inhibition of viral entry into epithelial cells and physical blocking of virus attachment. Intranasal vaccination against influenza is clinically established through live-attenuated influenza vaccines (LAIV), including FluMist/Fluenz. However, no antiviral nasal formulation for the treatment of influenza virus infection in humans is currently available on the market, highlighting the need for further research and development in this field.

The development of nasal drug formulations requires extensive preclinical evaluation before clinical testing can proceed. Many aspects of influenza virus infection, such as viral replication and early host responses can be investigated using human-relevant in vitro and ex vivo models. Readouts including viral titers, cytotoxicity, and cytokine responses provide valuable endpoints that can be measured in controlled experimental systems and may provide early indications of antiviral efficacy. Such approaches offer the opportunity to improve the relevance of experimental models to human physiology.

Based on the evidence discussed in this review, a stepwise translational framework, integrating in vitro, ex vivo, and in vivo models according to the specific biological question under investigation can support the efficient evaluation of antiviral nasal formulations. Initial studies should focus on identifying relevant endpoints that correlate with clinical outcomes in human influenza infection, thereby strengthening the translational relevance of preclinical models. Early screening can then be performed using two-dimensional in vitro assays to evaluate cytotoxicity, antiviral activity, and formulation compatibility. Subsequently, more advanced in vitro and ex vivo systems such as differentiated airway epithelial (ALI) cultures, nasal organoids, or organ-on-chip models can provide deeper insights into host-pathogen interactions and drug performance under conditions that better resemble the human respiratory epithelium. Critically, each biological model requires an administration or exposure system tailored to its geometry and physiology. Exposure or dosing protocols for ALI cultures, organoids, or organ-on-chip systems must therefore be adapted to the accessibility and architecture of the respective model. Matching the exposure system to the biological model and to the intended clinical route of administration is therefore an integral part of the translational framework. Animal models may still be required for specific questions that cannot yet be addressed in vitro or ex vivo, such as systemic pharmacokinetics or viral transmission. Their inclusion should be guided by the same principle applied to in vitro and ex vivo models, selection according to the specific biological question, with well-validated models, clearly defined endpoints, and justified use in line with reduction and refinement principles.

In summary, the development of antiviral nasal sprays for influenza therapy represents a promising yet underexplored strategy in antiviral drug delivery. By integrating in vitro, ex vivo, and in vivo models each paired with an appropriate exposure system and selected according to the biological question of interest, it is possible to streamline the development process, improve translational relevance, and reduce the reliance on animal experimentation. Such an integrated translational framework may accelerate the development of nasal antiviral formulations and ultimately facilitate the introduction of the first clinically approved nasal spray for the treatment of influenza virus infections.

## Data Availability

No primary data were generated in this study; all data are from published sources cited in the text.
